# Crystal structures of four chiral imine-substituted thio­phene derivatives

**DOI:** 10.1107/S2056989016002516

**Published:** 2016-02-17

**Authors:** Guadalupe Hernández-Téllez, Sylvain Bernès, Angel Mendoza, Francisco Javier Ríos-Merino, Gloria E. Moreno, Oscar Portillo, René Gutiérrez

**Affiliations:** a Laboratorio de Síntesis de Complejos, Facultad de Ciencias Químicas, Universidad Autónoma de Puebla, A.P. 1067, 72001 Puebla, Pue., Mexico; b Instituto de Física, Universidad Autónoma de Puebla, Av. San Claudio y 18 Sur, 72570 Puebla, Pue., Mexico; c Centro de Química, Instituto de Ciencias, Universidad Autónoma de Puebla, 72570 Puebla, Pue., Mexico

**Keywords:** crystal structure, Schiff base, bis-imine, thio­phene

## Abstract

A series of thio­phenes substituted in positions 2 and 5 by imine groups have been synthesized using a solvent-free approach, and their crystal structures determined. The substituents are chiral groups, and the expected absolute configuration for each mol­ecule was confirmed by refinement of the Flack parameter. The compounds are 2,5-bis­[(*S*)-(+)-(1,2,3,4-tetra­hydro­naphthalen-1-yl)imino]­thio­phene, C_26_H_26_N_2_S, (I), 2,5-bis­{[(*R*)-(−)-1-(4-meth­oxy­phen­yl)eth­yl]imino­meth­yl}thio­phene, C_24_H_26_N_2_O_2_S, (II), 2,5-bis­{[(*R*)-(−)-1-(4-fluoro­phen­yl)eth­yl]imino­meth­yl}thio­phene, C_22_H_20_F_2_N_2_S, (III), and 2,5-bis­{[(*S*)-(+)-1-(4-chloro­phen­yl)eth­yl]imino­meth­yl}thio­phene, C_22_H_20_Cl_2_N_2_S, (IV). A common feature of all four mol­ecules is the presence of twofold symmetry. For (I), which crystallizes in the triclinic space group *P*1, this symmetry is non-crystallographic, but for (II) in *C*2 and the isomorphous structures (III) and (IV) that crystallize in *P*2_1_2_1_2, the twofold symmetry is crystallographically imposed with one half of each mol­ecule in the asymmetric unit. The comparable mol­ecular symmetry in the four structures is also reflected in similar packing, with mol­ecules aggregated to form chains through weak C—H⋯S inter­actions.

## Abstract 2

Thio­phenes substituted in position 2 and 5 by chiral imine groups display non-crystallographic or crystallographic twofold symmetry.

## Chemical context   

Thio­phene­dicarbaldehydes have a variety of applications (Dean, 1982*a* ▸,*b* ▸), for instance in the synthesis of annulenones and polyenyl-substituted thio­phenes (Sargent & Cresp, 1975 ▸), in the preparation of macrocyclic ligands for bimetallic complexes that are able to mimic enzymes (Nelson *et al.*, 1983 ▸), in crown ether chemistry (Cram & Trueblood, 1981 ▸) and, more recently, in the preparation of azomethines for photovoltaic applications (Bolduc *et al.*, 2013*a* ▸,*b* ▸; Petrus *et al.*, 2014 ▸). In regard to this latter application, most of the conjugated materials used in organic electronics are synthesized using time-consuming Suzuki-, Wittig-, or Heck-type coupling reactions that require expensive catalysts, stringent reaction conditions, and tedious purification processes. In order to afford a more economic route towards organic photovoltaic materials, Schiff bases derived from 2,5-thio­phene­dicarbaldehyde as the conjugated linker unit have recently been used. The azomethine bond, which is isoelectronic with the vinyl bond and possesses similar optoelectronic and thermal properties, is easily accessible through the Schiff condensation under near ambient reaction conditions (Morgan *et al.*, 1987 ▸; Pérez Guarìn *et al.*, 2007 ▸; Sicard *et al.*, 2013 ▸).
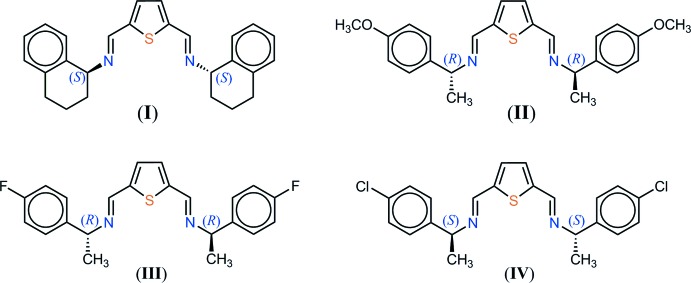


We report here the synthesis and X-ray characterization of such thio­phene derivatives, as a continuation of a partially published record (Bernès *et al.*, 2013 ▸; Mendoza *et al.*, 2014 ▸). We are improving a general solvent-free approach for these syntheses, recognising that ecological aspects in organic chemistry have become a priority, in order to minimize the qu­antity of toxic waste and by-products, and to decrease the amount of solvent in the reaction media or during work-up (Tanaka & Toda, 2000 ▸; Noyori, 2005 ▸).

In the synthesis of the thio­phenes reported here, the Schiff condensation generates a single by-product, water, and a one-step recrystallization affords the pure substituted thio­phene in nearly qu­anti­tative yields. Our protocol may be readily extended to any low mol­ecular weight 2,5-susbtituted thio­phene, providing that a liquid amine is used for the condensation. In the present work, the starting material is 2,5-thio­phene­dicarbaldehyde, a low melting-point compound (m.p. = 388–390 K), and four chiral amines were used. We took advantage of the anomalous dispersion of the sulfur sites to confirm that the configuration of the chiral amine is retained during the condensation.

## Structural commentary   

**Figure 1:**
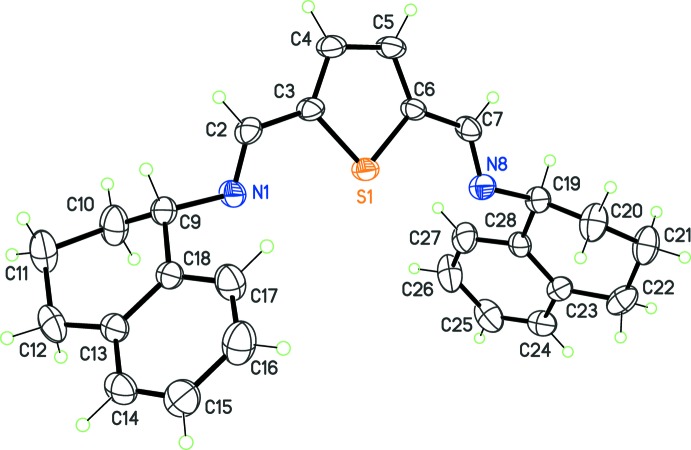
The mol­ecular structure of (I), with displacement ellipsoids for non-H atoms at the 30% probability level.

The first compound was synthesized using (*S*)-(+)-1-amino­tetra­line. The Schiff base (I), C_26_H_26_N_2_S, crystallizes in the space group *P*1, with the expected absolute configuration (Fig. 1 ▸). The general shape of the mol­ecule displays a pseudo-twofold axis, passing through the S atom and the midpoint of the thio­phene C—C σ-bond. As a consequence, the independent benzene rings are placed above and below the thio­phene ring, and are inclined to one another at a dihedral angle of 73.76 (15)°. The central core containing the thio­phene ring and the imine bonds is virtually planar, and the imine bonds are substituted by the tetra­lin ring systems, which present the same conformation. The aliphatic rings C9–C13/C18 and C19–C23/C28 each have a half-chair conformation.

**Figure 2:**
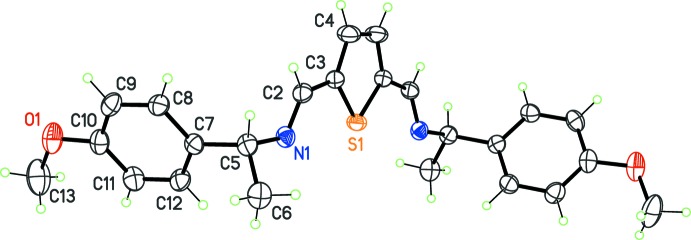
The mol­ecular structure of (II), with displacement ellipsoids for non-H atoms at the 30% probability level. Non-labeled atoms are generated by symmetry code (1 − *x*, *y*, 1 − *z*).

Compound (II), C_24_H_26_N_2_O_2_S, was obtained using (*R*)-(+)-(4-meth­oxy)phenyl­ethyl­amine as the chiral component in the Schiff condensation. The twofold mol­ecular axis, which was a latent symmetry in the case of (I), is a true crystallographic symmetry in (II), and this compound crystallizes in the space group *C*2 (Fig. 2 ▸). The asymmetric unit thus contains half a mol­ecule, and the mol­ecular conformation for the complete mol­ecule is similar to that of (I). The benzene rings have a free relative orientation, since these rings are not fused in a bicyclic system, as in (I); the dihedral angle between symmetry-related rings is 61.30 (7)°.

**Figure 3:**
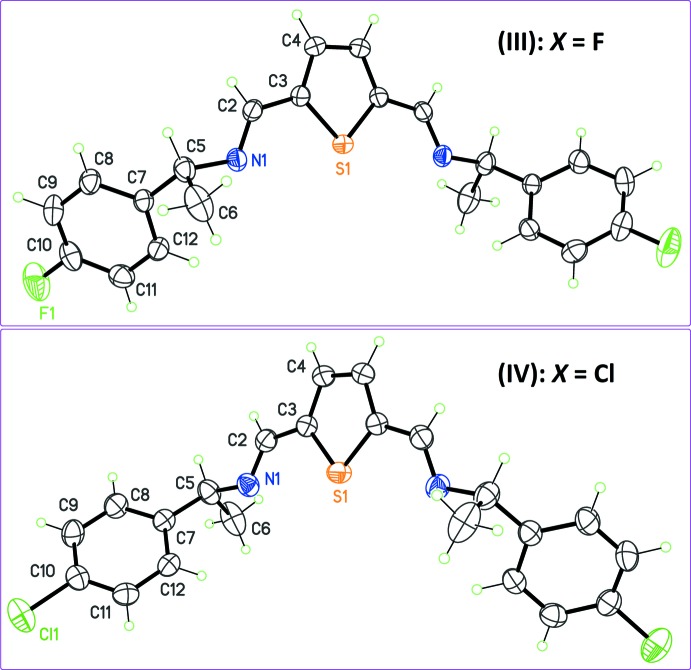
The mol­ecular structures of isomorphous compounds (III) and (IV), with displacement ellipsoids for non-H atoms at the 30% probability level. Notice the different configuration for chiral center C5 in (III) and (IV). Non-labeled atoms are generated by symmetry codes (1 − *x*, −*y*, *z*) and (1 − *x*, 2 − *y*, *z*) for (III) and (IV), respectively.

Compounds (III) and (IV), synthesized with enanti­o­meri­cally pure (4-halogen)phenyl­ethyl­amines (halogen = F, Cl) are isomorphous and crystallize with ortho­rhom­bic unit cells. The latent twofold symmetry of (I) is again observed, since both mol­ecules lie on the crystallographic twofold axes of the space group *P*2_1_2_1_2 (Fig. 3 ▸). The dihedral angle between the benzene rings is close to that observed for (II): 64.18 (8)° for (III) and 62.03 (9)° for (IV). The same Schiff base but with Br as the halogen substituent has been published previously (Mendoza *et al.*, 2014 ▸), but is not isomorphous with (III) and (IV). Instead, this mol­ecule was found to crystallize in the space group *C*2, with unit-cell parameters and a crystal structure very similar to those of (II). A systematic trend is thus emerging for these 2,5-substituted thio­phenes, related to the potential twofold mol­ecular symmetry: they have a strong tendency to crystallize in space groups that include at least one *C*_2_ axis, such as *C*2 and *P*2_1_2_1_2 for the chiral crystals. This trend extends to achiral mol­ecules, which also have twofold crystallographic symmetry in the space group *C*2/*c* (Kudyakova *et al.*, 2011 ▸; Suganya *et al.*, 2014 ▸; Boyle *et al.*, 2015 ▸; Moussallem *et al.*, 2015 ▸). The features shared by these related compounds could also be a signature of a propensity towards polymorphism between monoclinic and ortho­rhom­bic systems.

The difference between non-crystallographic symmetry in (I) and exact *C*_2_ mol­ecular symmetry in (II)–(IV) is also reflected in the degree of conjugation between thio­phene rings and imine bonds. For (I), dihedral angles between the thio­phene and C=N—C^*^ mean planes (C^*^ is the chiral C atom bonded to the imine functionality) are 6.9 (7) and 1.9 (6)°. Other crystals have a symmetry restriction, inducing a small deconjugation of the imine bonds. The corresponding dihedral angles with the thio­phene rings are 8.5 (4), 10.1 (3), and 9.8 (3)°, for (II), (III) and (IV), respectively.

## Supra­molecular features   

**Figure 4:**
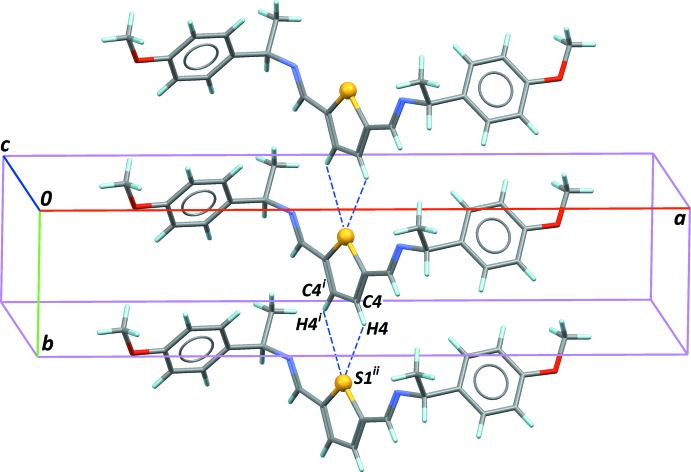
Part of the crystal structure of (II), showing C—H⋯S hydrogen bonds (dashed lines) linking mol­ecules along [010]. [Symmetry codes: (i) 1 − *x*, *y*, 1 − *z*; (ii) *x*, 1 + *y*, *z*.]

Although all compounds have benzene rings, neither π–π nor C—H⋯π contacts stabilize the crystal structures. However, these compounds share a common supra­molecular feature. Lone pairs of S atoms inter­act with thio­phenic CH groups of a neighboring mol­ecule in the crystal, forming chains along the short cell axes: [100] for (I), [010] for (II) and [001] for (III) and (IV). An example is presented in Fig. 4 ▸, for compound (II). These bifurcated S⋯C—H contacts have a significant strength for (I), perhaps as a consequence of the relaxed mol­ecular symmetry in space group *P*1. The contacts are weaker for (II), (III) and (IV), which have a geometry restrained by the crystallographic symmetry (Table 1 ▸).

## Database survey   

Many thio­phenes substituted in the 2 and 5 positions by imine groups have been characterized; however, almost all were achiral compounds. X-ray structures have been reported mostly in space group *C*2/*c* (Suganya *et al.*, 2014 ▸; Kudyakova *et al.*, 2011 ▸, 2012 ▸; Bolduc *et al.*, 2013*b* ▸). Other represented space groups for achiral mol­ecules are *P*2_1_ (Skene & Dufresne, 2006 ▸) and *P*2_1_/*c* (Wiedermann *et al.*, 2005 ▸). Finally, a single case of a mol­ecule presenting mirror symmetry has been described (Fridman & Kaftory, 2007 ▸), in space group *Pnma*.

The group of chiral mol­ecules belonging to this family is much less populated, with two examples reported by our group in this journal. Both are mol­ecules with the *C*_2_ point group and crystallize in space groups *C*2 (Mendoza *et al.*, 2014 ▸) and *P*22_1_2_1_ (Bernès *et al.*, 2013 ▸).

## Synthesis and crystallization   

**Synthesis**. The chiral amines used for the Schiff condensation were obtained directly from suppliers: (*S*)-(+)-1,2,3,4-tetra­hydro-1-naphthyl­amine for (I), (*R*)-(+)-1-(4-meth­oxy­phen­yl)ethyl­amine for (II), (*R*)-(+)-1-(4-fluoro­phen­yl)ethyl­amine for (III) and (*S*)-(−)-1-(4-chloro­phen­yl)ethyl­amine for (IV). 2,5-Thio­phene­dicarbaldehyde (100 mg, 0.71 mmol) and the chiral amine (1.4 mmol) in a 1:2 molar ratio were mixed at room temperature under solvent-free conditions, giving light-yellow (II and IV), colorless (III) or light-brown (IV) solids, in 95-97% yields. The crude solids were recrystallized from CH_2_Cl_2_, affording colorless crystals of (I)–(IV).

**Spectroscopy**. (I): m.p. 437–438 K. [α]^20^_D_ = +655.4 (*c* = 1, CHCl_3_). FTIR: 1616 cm^−1^ (C=N). ^1^H NMR (500 MHz, CHCl_3_/TMS): δ = 1.76–1.86 (*m*, 2H; *H*-al), 1.96–2.06 (*m*, 6H; *H*-al), 2.74–2.90 (*m*, 4H; *H*-al), 4.51 (*t*, 2H; *H*-al), 6.98–7.02 (*m*, 2H; *H*-ar), 7.09–7.15 (*m*, 6H; *H*-ar), 7.28 (*s*, 2H; *H*-ar), 8.36 (*s*, 2H; *H*C=N). ^13^C NMR: δ = 19.7, 29.3, 31.1, 67.7 (*C*-al), 125.7, 126.9, 128.7, 129.1, 129.6, 136.8, 137.1, 145.1 (*C*-ar), 153.1 (H*C*=N). MS–EI: *m*/*z* = 398 (*M* ^+^).

(II): m.p. 405–406 K. [α]^20^_D_ = −626.8 (*c* = 1, CHCl_3_). FTIR: 1631 cm^−1^ (C=N). ^1^H NMR (500 MHz, CHCl_3_/TMS): δ = 1.53 (*d*, 6H; CHC*H*_3_), 3.78 (*s*, 6H; OC*H*_3_), 4.47 (*q*, 2H; C*H*CH_3_), 6.85–6.88 (*m*, 4H; *H*-ar), 7.19 (*s*, 2H; *H*-ar), 7.29–7.32 (*m*, 4H; *H*-ar), 8.33 (*s*, 2H; *H*C=N). ^13^C NMR: δ = 24.8 (CH*C*H_3_), 55.2 (O*C*H_3_), 68.1 (*C*HCH_3_), 113.7, 127.6, 129.6, 137.1, 145.2, 152.1 (*C*-ar), 158.5 (H*C*=N). MS–EI: *m*/*z* = 406 (*M* ^+^).

(III): m.p. 420–421 K. [α]^20^_D_ = −542.5 (*c* = 1, CHCl_3_). FTIR: 1621 cm^−1^ (C=N). ^1^H NMR (500 MHz, CHCl_3_/TMS): δ = 1.53 (*d*, 6H; CHC*H*_3_), 4.49 (*q*, 2H; C*H*CH_3_), 7.00–7.38 (*m*, 10H; *H*-ar), 8.37 (*s*, 2H; *H*C=N). ^13^C NMR: δ = 25.2 (CH*C*H_3_), 68.7 (*C*HCH_3_), 115.2 (*d*, *J*_F-C_ = 21.2 Hz; *C*-ar), 128.1 (*d*, *J*_F-C_ = 8.7 Hz; *C*-ar), 130.1 (*C*-ar), 140.7 (*d*, *J*_F-C_ = 2.5 Hz; *C*-ar), 145.1 (*C*-ar), 161.1 (*d*, *J*_F-C_ = 242.5 Hz; *C*-ar), 152.5 (H*C*=N). MS–EI: *m*/*z* = 382 (*M* ^+^).

(IV): m.p. 434–435 K. [α]^20^_D_ = +726.5 (*c* = 1, CHCl_3_). FTIR: 1623 cm^−1^ (C=N). ^1^H NMR (500 MHz, CHCl_3_/TMS): δ = 1.53 (*d*, 6H; CHC*H*_3_), 4.48 (*q*, 2H; C*H*CH_3_), 7.23–7.35 (*m*, 10H; *H*-ar), 8.37 (*s*, 2H; *H*C=N). ^13^C NMR: δ = 25.2 (CH*C*H_3_), 68.7 (*C*HCH_3_), 128.0, 128.6, 130.2, 132.5, 143.5, 145.1 (*C*-ar), 152.7 (H*C*=N).

## Refinement   

Crystal data, data collection and structure refinement details are summarized in Table 2 ▸. No unusual issues appeared, and refinements were carried out on non-restricted models. All H atoms were placed in calculated positions, and refined as riding on their carrier C atoms, with C—H bond lengths fixed to 0.93 (aromatic CH), 0.96 (methyl CH_3_), 0.97 (methyl­ene CH_2_), or 0.98 Å (methine CH). Isotropic displacement parameters were calculated as *U*_iso_(H) = 1.5*U*_eq_(C) for methyl H atoms and *U*_iso_(H) = 1.2*U*_eq_(C) for other H atoms. For all compounds, the absolute configuration was based on the refinement of the Flack parameter (Parsons *et al.*, 2013 ▸), confirming that the configuration of the chiral amine used as the starting material was retained during the Schiff condensation.

## Supplementary Material

Crystal structure: contains datablock(s) I, II, III, IV, global. DOI: 10.1107/S2056989016002516/sj5495sup1.cif

Structure factors: contains datablock(s) I. DOI: 10.1107/S2056989016002516/sj5495Isup2.hkl

Structure factors: contains datablock(s) II. DOI: 10.1107/S2056989016002516/sj5495IIsup3.hkl

Structure factors: contains datablock(s) III. DOI: 10.1107/S2056989016002516/sj5495IIIsup4.hkl

Structure factors: contains datablock(s) IV. DOI: 10.1107/S2056989016002516/sj5495IVsup5.hkl

Supporting information file. DOI: 10.1107/S2056989016002516/sj5495Isup6.cml

Supporting information file. DOI: 10.1107/S2056989016002516/sj5495IIsup7.cml

Supporting information file. DOI: 10.1107/S2056989016002516/sj5495IIIsup8.cml

Supporting information file. DOI: 10.1107/S2056989016002516/sj5495IVsup9.cml

CCDC references: 1452795, 1452794, 1452793, 1452792

Additional supporting information:  crystallographic information; 3D view; checkCIF report

## Figures and Tables

**Table 1:** Comparison of C—H⋯S hydrogen bonds (Å, °) in compounds (I)–(IV)

Compound	Contact	C—H	H⋯S	C⋯S	C—H⋯S
(I)	C4—H4*A*⋯S1^i^	0.93	3.00	3.562 (5)	121
(I)	C5—H5*A*⋯S1^i^	0.93	2.97	3.547 (5)	122
					
(II)	C4—H4*A*⋯S1^ii^	0.93	2.99	3.572 (3)	122
(III)	C4—H4*A*⋯S1^iii^	0.93	3.15	3.743 (3)	124
(IV)	C4—H4*A*⋯S1^iv^	0.93	3.23	3.828 (4)	124

Symmetry codes: (i) *x* + 1, *y*, *z*; (ii) *x*, *y* + 1, *z*; (iii) *x*, *y*, *z* + 1; (iv) *x*, *y*, *z* − 1.

**Table 2:** Experimental details

	(I)	(II)	(III)	(IV)
Crystal data				
Chemical formula	C_26_H_26_N_2_S	C_24_H_26_N_2_O_2_S	C_22_H_20_F_2_N_2_S	C_22_H_20_Cl_2_N_2_S
*M*_r_	398.55	406.53	382.46	415.36
Crystal system, space group	Triclinic, *P*1	Monoclinic, *C*2	Orthorhombic, *P*2_1_2_1_2	Orthorhombic, *P*2_1_2_1_2
Temperature (K)	298	298	298	298
*a*, *b*, *c* (Å)	5.9093 (4), 7.6258 (5), 12.6570 (8)	25.3917 (13), 5.9488 (3), 7.5623 (4)	21.1153 (16), 7.7846 (6), 6.1343 (5)	21.893 (2), 7.9212 (6), 6.2315 (4)
α, β, γ (°)	87.802 (5), 78.329 (5), 87.427 (5)	90, 97.174 (4), 90	90, 90, 90	90, 90, 90
*V* (Å^3^)	557.76 (6)	1133.34 (10)	1008.32 (14)	1080.66 (15)
*Z*	1	2	2	2
Radiation type	Mo *K*α	Mo *K*α	Mo *K*α	Mo *K*α
μ (mm^−1^)	0.16	0.16	0.19	0.41
Crystal size (mm)	0.34 × 0.12 × 0.06	0.45 × 0.33 × 0.12	0.89 × 0.47 × 0.33	0.52 × 0.40 × 0.07

Data collection				
Diffractometer	Agilent Xcalibur (Atlas, Gemini)	Agilent Xcalibur (Atlas, Gemini)	Agilent Xcalibur (Atlas, Gemini)	Agilent Xcalibur (Atlas, Gemini)
Absorption correction	Analytical *CrysAlis PRO*, (Agilent, 2013 ▸)	Analytical (*CrysAlis PRO*; Agilent, 2013 ▸)	Analytical *CrysAlis PRO*, (Agilent, 2013 ▸)	Multi-scan *CrysAlis PRO*, (Agilent, 2013 ▸)
*T*_min_, *T*_max_	0.969, 0.992	0.973, 0.993	0.904, 0.958	0.692, 1.000
No. of measured, independent and observed [*I* > 2σ(*I*)] reflections	6689, 4036, 2958	6341, 2221, 1892	12336, 2067, 1591	14195, 2743, 1534
*R*_int_	0.040	0.027	0.058	0.058
(sin θ/λ)_max_ (Å^−1^)	0.618	0.618	0.625	0.692

Refinement				
*R*[*F* ^2^ > 2σ(*F* ^2^)], *wR*(*F* ^2^), *S*	0.058, 0.127, 1.02	0.036, 0.085, 1.02	0.044, 0.092, 1.06	0.052, 0.117, 1.01
No. of reflections	4036	2221	2067	2743
No. of parameters	262	134	124	124
No. of restraints	3	1	0	0
H-atom treatment	H-atom parameters constrained	H-atom parameters constrained	H-atom parameters constrained	H-atom parameters constrained
Δρ_max_, Δρ_min_ (e Å^−3^)	0.31, −0.19	0.11, −0.17	0.15, −0.25	0.13, −0.17
Absolute structure	Flack *x* determined using 962 quotients [(*I* ^+^)−(*I* ^−^)]/[(*I* ^+^)+(*I* ^−^)] (Parsons *et al.*, 2013 ▸)	Flack *x* determined using 708 quotients [(*I* ^+^)−(*I* ^−^)]/[(*I* ^+^)+(*I* ^−^)] (Parsons *et al.*, 2013 ▸)	Flack *x* determined using 518 quotients [(*I* ^+^)−(*I* ^−^)]/[(*I* ^+^)+(*I* ^−^)] (Parsons *et al.*, 2013 ▸)	Flack *x* determined using 465 quotients [(*I* ^+^)−(*I* ^−^)]/[(*I* ^+^)+(*I* ^−^)] (Parsons *et al.*, 2013 ▸)
Absolute structure parameter	−0.12 (7)	−0.02 (4)	0.07 (6)	0.10 (6)

Computer programs: *CrysAlis PRO* (Agilent, 2013 ▸), *SHELXS97* (Sheldrick, 2008 ▸), *SHELXT* (Sheldrick, 2015*a* ▸), *SHELXL2014* (Sheldrick, 2015*b* ▸) and *Mercury* (Macrae *et al.*, 2008 ▸).

## supplementary crystallographic information

### (I) 2,5-Bis[(S)-(+)-(1,2,3,4-tetrahydro-1-naphthyl)imino]thiophene Crystal data

**Table:**

C_26_H_26_N_2_S	*F*(000) = 212
*M**_r_* = 398.55	*D*_x_ = 1.187 Mg m^−^^3^
Triclinic, *P*1	Melting point: 437 K
*a* = 5.9093 (4) Å	Mo *K*α radiation, λ = 0.71073 Å
*b* = 7.6258 (5) Å	Cell parameters from 2148 reflections
*c* = 12.6570 (8) Å	θ = 3.3–22.6°
α = 87.802 (5)°	µ = 0.16 mm^−^^1^
β = 78.329 (5)°	*T* = 298 K
γ = 87.427 (5)°	Plate, colorless
*V* = 557.76 (6) Å^3^	0.34 × 0.12 × 0.06 mm
*Z* = 1	

### (I) 2,5-Bis[(S)-(+)-(1,2,3,4-tetrahydro-1-naphthyl)imino]thiophene Data collection

**Table:**

Agilent Xcalibur (Atlas, Gemini) diffractometer	4036 independent reflections
Radiation source: Enhance (Mo) X-ray Source	2958 reflections with *I* > 2σ(*I*)
Graphite monochromator	*R*_int_ = 0.040
Detector resolution: 10.5564 pixels mm^-1^	θ_max_ = 26.1°, θ_min_ = 3.1°
ω scans	*h* = −7→7
Absorption correction: analytical *CrysAlis PRO*, (Agilent, 2013)	*k* = −9→9
*T*_min_ = 0.969, *T*_max_ = 0.992	*l* = −15→15
6689 measured reflections	

### (I) 2,5-Bis[(S)-(+)-(1,2,3,4-tetrahydro-1-naphthyl)imino]thiophene Refinement

**Table:**

Refinement on *F*^2^	Secondary atom site location: difference Fourier map
Least-squares matrix: full	Hydrogen site location: inferred from neighbouring sites
*R*[*F*^2^ > 2σ(*F*^2^)] = 0.058	H-atom parameters constrained
*wR*(*F*^2^) = 0.127	*w* = 1/[σ^2^(*F*_o_^2^) + (0.0525*P*)^2^] where *P* = (*F*_o_^2^ + 2*F*_c_^2^)/3
*S* = 1.02	(Δ/σ)_max_ < 0.001
4036 reflections	Δρ_max_ = 0.31 e Å^−^^3^
262 parameters	Δρ_min_ = −0.19 e Å^−^^3^
3 restraints	Absolute structure: Flack *x* determined using 962 quotients [(*I*^+^)-(*I*^-^)]/[(*I*^+^)+(*I*^-^)] (Parsons *et al.*, 2013)
0 constraints	Absolute structure parameter: −0.12 (7)
Primary atom site location: structure-invariant direct methods	

### (I) 2,5-Bis[(S)-(+)-(1,2,3,4-tetrahydro-1-naphthyl)imino]thiophene Fractional atomic coordinates and isotropic or equivalent isotropic displacement parameters (Å^2^)

**Table:**

	*x*	*y*	*z*	*U*_iso_*/*U*_eq_	
S1	0.66581 (19)	0.49640 (17)	0.11819 (12)	0.0488 (4)	
N1	0.5097 (7)	0.7980 (6)	0.2625 (3)	0.0507 (12)	
C2	0.7239 (10)	0.7657 (7)	0.2474 (4)	0.0490 (13)	
H2A	0.8132	0.8300	0.2834	0.059*	
C3	0.8355 (8)	0.6297 (7)	0.1747 (4)	0.0469 (13)	
C4	1.0622 (9)	0.5892 (7)	0.1402 (5)	0.0575 (15)	
H4A	1.1792	0.6461	0.1628	0.069*	
C5	1.1042 (8)	0.4510 (7)	0.0658 (5)	0.0595 (15)	
H5A	1.2513	0.4075	0.0348	0.071*	
C6	0.9068 (8)	0.3894 (6)	0.0450 (4)	0.0425 (12)	
C7	0.8786 (9)	0.2528 (7)	−0.0268 (4)	0.0503 (14)	
H7A	1.0094	0.1943	−0.0651	0.060*	
N8	0.6816 (8)	0.2106 (6)	−0.0390 (3)	0.0518 (12)	
C9	0.4190 (9)	0.9453 (7)	0.3325 (4)	0.0518 (13)	
H9A	0.5365	0.9745	0.3730	0.062*	
C10	0.3728 (12)	1.1032 (8)	0.2631 (5)	0.0772 (18)	
H10A	0.2751	1.0713	0.2143	0.093*	
H10B	0.5174	1.1431	0.2201	0.093*	
C11	0.2537 (13)	1.2501 (8)	0.3345 (5)	0.0802 (19)	
H11A	0.3449	1.2749	0.3875	0.096*	
H11B	0.2407	1.3560	0.2909	0.096*	
C12	0.0161 (11)	1.1958 (8)	0.3911 (5)	0.0682 (18)	
H12A	−0.0468	1.2796	0.4462	0.082*	
H12B	−0.0847	1.1991	0.3393	0.082*	
C13	0.0174 (9)	1.0143 (7)	0.4429 (4)	0.0486 (14)	
C14	−0.1721 (10)	0.9610 (9)	0.5196 (5)	0.0620 (16)	
H14A	−0.2950	1.0410	0.5406	0.074*	
C15	−0.1846 (11)	0.7955 (9)	0.5651 (5)	0.0749 (18)	
H15A	−0.3143	0.7635	0.6159	0.090*	
C16	−0.0009 (13)	0.6756 (9)	0.5347 (6)	0.080 (2)	
H16A	−0.0068	0.5621	0.5644	0.095*	
C17	0.1892 (11)	0.7268 (8)	0.4602 (5)	0.0665 (16)	
H17A	0.3134	0.6471	0.4414	0.080*	
C18	0.2020 (8)	0.8935 (7)	0.4123 (4)	0.0465 (12)	
C19	0.6721 (9)	0.0655 (6)	−0.1121 (4)	0.0498 (13)	
H19A	0.8294	0.0400	−0.1523	0.060*	
C20	0.5911 (13)	−0.0955 (8)	−0.0465 (5)	0.0728 (17)	
H20A	0.4515	−0.0668	0.0058	0.087*	
H20B	0.7086	−0.1390	−0.0075	0.087*	
C21	0.5425 (13)	−0.2380 (8)	−0.1206 (5)	0.0755 (19)	
H21A	0.6802	−0.2628	−0.1750	0.091*	
H21B	0.5024	−0.3453	−0.0786	0.091*	
C22	0.3465 (11)	−0.1769 (9)	−0.1746 (5)	0.0688 (18)	
H22A	0.3350	−0.2584	−0.2300	0.083*	
H22B	0.2028	−0.1782	−0.1216	0.083*	
C23	0.3768 (9)	0.0051 (8)	−0.2248 (4)	0.0503 (14)	
C24	0.2515 (10)	0.0601 (9)	−0.3022 (4)	0.0624 (15)	
H24A	0.1532	−0.0175	−0.3233	0.075*	
C25	0.2684 (12)	0.2252 (10)	−0.3484 (5)	0.079 (2)	
H25A	0.1830	0.2591	−0.4004	0.095*	
C26	0.4143 (14)	0.3418 (9)	−0.3167 (6)	0.086 (2)	
H26A	0.4269	0.4550	−0.3469	0.103*	
C27	0.5398 (11)	0.2877 (8)	−0.2403 (5)	0.0671 (17)	
H27A	0.6391	0.3653	−0.2199	0.080*	
C28	0.5226 (8)	0.1213 (7)	−0.1928 (4)	0.0484 (13)	

### (I) 2,5-Bis[(S)-(+)-(1,2,3,4-tetrahydro-1-naphthyl)imino]thiophene Atomic displacement parameters (Å^2^)

**Table:**

	*U*^11^	*U*^22^	*U*^33^	*U*^12^	*U*^13^	*U*^23^
S1	0.0351 (7)	0.0574 (8)	0.0551 (7)	−0.0009 (5)	−0.0088 (5)	−0.0201 (6)
N1	0.045 (3)	0.058 (3)	0.049 (3)	0.004 (2)	−0.006 (2)	−0.026 (2)
C2	0.049 (3)	0.055 (3)	0.047 (3)	−0.007 (3)	−0.013 (2)	−0.016 (3)
C3	0.039 (3)	0.058 (3)	0.048 (3)	−0.002 (2)	−0.016 (2)	−0.014 (3)
C4	0.036 (3)	0.071 (4)	0.070 (4)	0.000 (3)	−0.017 (3)	−0.027 (3)
C5	0.033 (3)	0.075 (4)	0.072 (4)	0.005 (3)	−0.010 (2)	−0.031 (3)
C6	0.031 (3)	0.050 (3)	0.046 (3)	0.004 (2)	−0.006 (2)	−0.011 (2)
C7	0.046 (3)	0.057 (3)	0.048 (3)	0.010 (3)	−0.007 (2)	−0.018 (3)
N8	0.048 (3)	0.059 (3)	0.050 (3)	−0.003 (2)	−0.008 (2)	−0.023 (2)
C9	0.049 (3)	0.053 (3)	0.054 (3)	−0.001 (3)	−0.009 (3)	−0.022 (3)
C10	0.102 (5)	0.061 (4)	0.060 (4)	−0.002 (3)	0.005 (3)	−0.015 (3)
C11	0.109 (5)	0.050 (4)	0.070 (4)	0.005 (3)	0.010 (4)	−0.007 (3)
C12	0.082 (5)	0.062 (4)	0.060 (4)	0.024 (3)	−0.015 (3)	−0.022 (3)
C13	0.048 (3)	0.057 (3)	0.043 (3)	0.002 (3)	−0.012 (3)	−0.016 (3)
C14	0.056 (3)	0.075 (4)	0.056 (3)	0.009 (3)	−0.009 (3)	−0.027 (3)
C15	0.075 (4)	0.082 (5)	0.062 (4)	−0.011 (4)	0.006 (3)	−0.022 (4)
C16	0.104 (5)	0.061 (4)	0.066 (4)	−0.012 (4)	0.001 (4)	−0.002 (4)
C17	0.072 (4)	0.059 (4)	0.064 (4)	0.007 (3)	−0.003 (3)	−0.011 (3)
C18	0.045 (3)	0.054 (3)	0.043 (3)	0.001 (3)	−0.012 (2)	−0.019 (3)
C19	0.052 (3)	0.053 (3)	0.044 (3)	0.000 (3)	−0.007 (2)	−0.017 (3)
C20	0.114 (5)	0.060 (4)	0.051 (3)	−0.010 (4)	−0.030 (3)	−0.011 (3)
C21	0.119 (5)	0.055 (4)	0.056 (4)	−0.012 (4)	−0.021 (4)	−0.009 (3)
C22	0.074 (4)	0.078 (5)	0.056 (4)	−0.028 (4)	−0.008 (3)	−0.017 (3)
C23	0.048 (3)	0.057 (3)	0.043 (3)	−0.007 (3)	0.001 (3)	−0.019 (3)
C24	0.056 (3)	0.079 (4)	0.054 (3)	0.003 (3)	−0.013 (3)	−0.028 (3)
C25	0.096 (5)	0.084 (5)	0.066 (4)	0.026 (4)	−0.037 (4)	−0.028 (4)
C26	0.129 (6)	0.057 (4)	0.080 (5)	0.016 (4)	−0.044 (5)	−0.010 (4)
C27	0.084 (4)	0.059 (4)	0.064 (4)	−0.003 (3)	−0.022 (4)	−0.019 (3)
C28	0.050 (3)	0.046 (3)	0.049 (3)	0.006 (2)	−0.006 (2)	−0.018 (3)

### (I) 2,5-Bis[(S)-(+)-(1,2,3,4-tetrahydro-1-naphthyl)imino]thiophene Geometric parameters (Å, º)

**Table:**

S1—C6	1.724 (5)	C14—H14A	0.9300
S1—C3	1.728 (5)	C15—C16	1.390 (9)
N1—C2	1.255 (6)	C15—H15A	0.9300
N1—C9	1.471 (6)	C16—C17	1.373 (9)
C2—C3	1.458 (7)	C16—H16A	0.9300
C2—H2A	0.9300	C17—C18	1.386 (8)
C3—C4	1.348 (7)	C17—H17A	0.9300
C4—C5	1.420 (7)	C19—C20	1.496 (7)
C4—H4A	0.9300	C19—C28	1.518 (7)
C5—C6	1.355 (6)	C19—H19A	0.9800
C5—H5A	0.9300	C20—C21	1.536 (8)
C6—C7	1.445 (7)	C20—H20A	0.9700
C7—N8	1.263 (6)	C20—H20B	0.9700
C7—H7A	0.9300	C21—C22	1.508 (9)
N8—C19	1.479 (6)	C21—H21A	0.9700
C9—C10	1.512 (8)	C21—H21B	0.9700
C9—C18	1.520 (7)	C22—C23	1.507 (9)
C9—H9A	0.9800	C22—H22A	0.9700
C10—C11	1.522 (8)	C22—H22B	0.9700
C10—H10A	0.9700	C23—C24	1.384 (8)
C10—H10B	0.9700	C23—C28	1.389 (7)
C11—C12	1.510 (9)	C24—C25	1.367 (9)
C11—H11A	0.9700	C24—H24A	0.9300
C11—H11B	0.9700	C25—C26	1.390 (10)
C12—C13	1.509 (8)	C25—H25A	0.9300
C12—H12A	0.9700	C26—C27	1.374 (8)
C12—H12B	0.9700	C26—H26A	0.9300
C13—C14	1.390 (8)	C27—C28	1.382 (7)
C13—C18	1.398 (7)	C27—H27A	0.9300
C14—C15	1.366 (9)		
			
C6—S1—C3	91.5 (2)	C16—C15—H15A	120.4
C2—N1—C9	116.5 (4)	C17—C16—C15	119.2 (7)
N1—C2—C3	121.5 (5)	C17—C16—H16A	120.4
N1—C2—H2A	119.3	C15—C16—H16A	120.4
C3—C2—H2A	119.3	C16—C17—C18	122.1 (6)
C4—C3—C2	129.7 (5)	C16—C17—H17A	119.0
C4—C3—S1	111.3 (4)	C18—C17—H17A	119.0
C2—C3—S1	119.1 (4)	C17—C18—C13	118.7 (5)
C3—C4—C5	113.2 (5)	C17—C18—C9	120.0 (5)
C3—C4—H4A	123.4	C13—C18—C9	121.2 (5)
C5—C4—H4A	123.4	N8—C19—C20	109.4 (4)
C6—C5—C4	112.6 (5)	N8—C19—C28	110.1 (4)
C6—C5—H5A	123.7	C20—C19—C28	113.3 (4)
C4—C5—H5A	123.7	N8—C19—H19A	108.0
C5—C6—C7	129.0 (5)	C20—C19—H19A	108.0
C5—C6—S1	111.4 (4)	C28—C19—H19A	108.0
C7—C6—S1	119.6 (4)	C19—C20—C21	109.9 (4)
N8—C7—C6	121.9 (5)	C19—C20—H20A	109.7
N8—C7—H7A	119.1	C21—C20—H20A	109.7
C6—C7—H7A	119.1	C19—C20—H20B	109.7
C7—N8—C19	117.5 (4)	C21—C20—H20B	109.7
N1—C9—C10	109.1 (4)	H20A—C20—H20B	108.2
N1—C9—C18	110.3 (4)	C22—C21—C20	109.9 (5)
C10—C9—C18	111.6 (5)	C22—C21—H21A	109.7
N1—C9—H9A	108.6	C20—C21—H21A	109.7
C10—C9—H9A	108.6	C22—C21—H21B	109.7
C18—C9—H9A	108.6	C20—C21—H21B	109.7
C9—C10—C11	109.6 (5)	H21A—C21—H21B	108.2
C9—C10—H10A	109.7	C23—C22—C21	112.9 (5)
C11—C10—H10A	109.7	C23—C22—H22A	109.0
C9—C10—H10B	109.7	C21—C22—H22A	109.0
C11—C10—H10B	109.7	C23—C22—H22B	109.0
H10A—C10—H10B	108.2	C21—C22—H22B	109.0
C12—C11—C10	109.6 (5)	H22A—C22—H22B	107.8
C12—C11—H11A	109.7	C24—C23—C28	119.1 (5)
C10—C11—H11A	109.7	C24—C23—C22	119.5 (5)
C12—C11—H11B	109.7	C28—C23—C22	121.4 (5)
C10—C11—H11B	109.7	C25—C24—C23	121.8 (6)
H11A—C11—H11B	108.2	C25—C24—H24A	119.1
C13—C12—C11	112.9 (5)	C23—C24—H24A	119.1
C13—C12—H12A	109.0	C24—C25—C26	119.3 (6)
C11—C12—H12A	109.0	C24—C25—H25A	120.4
C13—C12—H12B	109.0	C26—C25—H25A	120.4
C11—C12—H12B	109.0	C27—C26—C25	119.1 (6)
H12A—C12—H12B	107.8	C27—C26—H26A	120.4
C14—C13—C18	118.4 (5)	C25—C26—H26A	120.4
C14—C13—C12	120.1 (5)	C26—C27—C28	121.9 (6)
C18—C13—C12	121.5 (5)	C26—C27—H27A	119.1
C15—C14—C13	122.5 (6)	C28—C27—H27A	119.1
C15—C14—H14A	118.8	C27—C28—C23	118.8 (5)
C13—C14—H14A	118.8	C27—C28—C19	119.8 (5)
C14—C15—C16	119.1 (6)	C23—C28—C19	121.3 (5)
C14—C15—H15A	120.4		
			
C9—N1—C2—C3	−176.4 (5)	C12—C13—C18—C17	−177.8 (5)
N1—C2—C3—C4	172.4 (6)	C14—C13—C18—C9	−177.3 (5)
N1—C2—C3—S1	−6.2 (7)	C12—C13—C18—C9	5.4 (7)
C6—S1—C3—C4	−1.4 (5)	N1—C9—C18—C17	39.8 (6)
C6—S1—C3—C2	177.5 (4)	C10—C9—C18—C17	161.1 (5)
C2—C3—C4—C5	−177.8 (5)	N1—C9—C18—C13	−143.5 (4)
S1—C3—C4—C5	0.9 (6)	C10—C9—C18—C13	−22.1 (6)
C3—C4—C5—C6	0.2 (7)	C7—N8—C19—C20	105.5 (6)
C4—C5—C6—C7	178.9 (5)	C7—N8—C19—C28	−129.5 (5)
C4—C5—C6—S1	−1.3 (6)	N8—C19—C20—C21	170.8 (5)
C3—S1—C6—C5	1.5 (4)	C28—C19—C20—C21	47.7 (7)
C3—S1—C6—C7	−178.7 (4)	C19—C20—C21—C22	−64.0 (7)
C5—C6—C7—N8	−179.1 (6)	C20—C21—C22—C23	49.0 (7)
S1—C6—C7—N8	1.1 (7)	C21—C22—C23—C24	161.6 (5)
C6—C7—N8—C19	−178.2 (5)	C21—C22—C23—C28	−20.5 (8)
C2—N1—C9—C10	102.3 (6)	C28—C23—C24—C25	0.4 (8)
C2—N1—C9—C18	−134.9 (5)	C22—C23—C24—C25	178.4 (6)
N1—C9—C10—C11	173.7 (5)	C23—C24—C25—C26	−0.3 (9)
C18—C9—C10—C11	51.6 (7)	C24—C25—C26—C27	0.5 (10)
C9—C10—C11—C12	−66.2 (7)	C25—C26—C27—C28	−0.9 (10)
C10—C11—C12—C13	48.1 (7)	C26—C27—C28—C23	1.0 (8)
C11—C12—C13—C14	164.1 (5)	C26—C27—C28—C19	177.5 (6)
C11—C12—C13—C18	−18.7 (8)	C24—C23—C28—C27	−0.7 (7)
C18—C13—C14—C15	−0.5 (8)	C22—C23—C28—C27	−178.7 (6)
C12—C13—C14—C15	176.8 (5)	C24—C23—C28—C19	−177.2 (5)
C13—C14—C15—C16	0.5 (9)	C22—C23—C28—C19	4.9 (8)
C14—C15—C16—C17	0.5 (10)	N8—C19—C28—C27	41.7 (6)
C15—C16—C17—C18	−1.6 (10)	C20—C19—C28—C27	164.5 (5)
C16—C17—C18—C13	1.6 (9)	N8—C19—C28—C23	−141.9 (5)
C16—C17—C18—C9	178.4 (6)	C20—C19—C28—C23	−19.1 (7)
C14—C13—C18—C17	−0.5 (7)		

### (I) 2,5-Bis[(S)-(+)-(1,2,3,4-tetrahydro-1-naphthyl)imino]thiophene Hydrogen-bond geometry (Å, º)

**Table:**

*D*—H···*A*	*D*—H	H···*A*	*D*···*A*	*D*—H···*A*
C4—H4*A*···S1^i^	0.93	3.00	3.562 (5)	121
C5—H5*A*···S1^i^	0.93	2.97	3.547 (5)	122

Symmetry code: (i) *x*+1, *y*, *z*.

### (II) 2,5-Bis{[(R)-(-)-1-(4-methoxyphenyl)ethyl]iminomethyl}thiophene Crystal data

**Table:**

C_24_H_26_N_2_O_2_S	*D*_x_ = 1.191 Mg m^−^^3^
*M**_r_* = 406.53	Melting point: 405 K
Monoclinic, *C*2	Mo *K*α radiation, λ = 0.71073 Å
*a* = 25.3917 (13) Å	Cell parameters from 2504 reflections
*b* = 5.9488 (3) Å	θ = 3.0–24.2°
*c* = 7.5623 (4) Å	µ = 0.16 mm^−^^1^
β = 97.174 (4)°	*T* = 298 K
*V* = 1133.34 (10) Å^3^	Prism, colourless
*Z* = 2	0.45 × 0.33 × 0.12 mm
*F*(000) = 432	

### (II) 2,5-Bis{[(R)-(-)-1-(4-methoxyphenyl)ethyl]iminomethyl}thiophene Data collection

**Table:**

Agilent Xcalibur (Atlas, Gemini) diffractometer	2221 independent reflections
Radiation source: Enhance (Mo) X-ray Source	1892 reflections with *I* > 2σ(*I*)
Graphite monochromator	*R*_int_ = 0.027
ω scans	θ_max_ = 26.1°, θ_min_ = 3.0°
Absorption correction: analytical (*CrysAlis PRO*; Agilent, 2013)	*h* = −31→31
*T*_min_ = 0.973, *T*_max_ = 0.993	*k* = −7→7
6341 measured reflections	*l* = −9→9

### (II) 2,5-Bis{[(R)-(-)-1-(4-methoxyphenyl)ethyl]iminomethyl}thiophene Refinement

**Table:**

Refinement on *F*^2^	Hydrogen site location: inferred from neighbouring sites
Least-squares matrix: full	H-atom parameters constrained
*R*[*F*^2^ > 2σ(*F*^2^)] = 0.036	*w* = 1/[σ^2^(*F*_o_^2^) + (0.0393*P*)^2^ + 0.1801*P*] where *P* = (*F*_o_^2^ + 2*F*_c_^2^)/3
*wR*(*F*^2^) = 0.085	(Δ/σ)_max_ < 0.001
*S* = 1.02	Δρ_max_ = 0.11 e Å^−^^3^
2221 reflections	Δρ_min_ = −0.17 e Å^−^^3^
134 parameters	Absolute structure: Flack *x* determined using 708 quotients [(*I*^+^)-(*I*^-^)]/[(*I*^+^)+(*I*^-^)] (Parsons *et al.*, 2013)
1 restraint	Absolute structure parameter: −0.02 (4)

### (II) 2,5-Bis{[(R)-(-)-1-(4-methoxyphenyl)ethyl]iminomethyl}thiophene Fractional atomic coordinates and isotropic or equivalent isotropic displacement parameters (Å^2^)

**Table:**

	*x*	*y*	*z*	*U*_iso_*/*U*_eq_	
S1	0.5000	0.37429 (14)	0.5000	0.0490 (3)	
N1	0.56565 (9)	0.3213 (4)	0.1855 (3)	0.0471 (6)	
C2	0.55195 (11)	0.5176 (5)	0.2189 (4)	0.0469 (7)	
H2A	0.5608	0.6324	0.1445	0.056*	
C3	0.52314 (10)	0.5774 (5)	0.3665 (4)	0.0449 (6)	
C4	0.51313 (13)	0.7856 (5)	0.4229 (4)	0.0596 (8)	
H4A	0.5225	0.9159	0.3665	0.072*	
C5	0.59848 (11)	0.2933 (4)	0.0386 (4)	0.0484 (7)	
H5A	0.5949	0.4291	−0.0354	0.058*	
C6	0.57863 (13)	0.0963 (6)	−0.0754 (4)	0.0658 (8)	
H6A	0.5416	0.1162	−0.1164	0.099*	
H6B	0.5835	−0.0394	−0.0065	0.099*	
H6C	0.5981	0.0861	−0.1759	0.099*	
C7	0.65613 (11)	0.2719 (4)	0.1189 (3)	0.0441 (6)	
C8	0.69277 (11)	0.4349 (4)	0.0871 (4)	0.0494 (7)	
H8A	0.6817	0.5585	0.0168	0.059*	
C9	0.74515 (11)	0.4171 (5)	0.1576 (4)	0.0571 (8)	
H9A	0.7692	0.5278	0.1343	0.069*	
C10	0.76215 (11)	0.2354 (6)	0.2628 (4)	0.0546 (7)	
C11	0.72642 (12)	0.0745 (6)	0.2994 (4)	0.0593 (8)	
H11A	0.7375	−0.0469	0.3722	0.071*	
C12	0.67374 (12)	0.0936 (6)	0.2275 (4)	0.0551 (8)	
H12A	0.6497	−0.0161	0.2528	0.066*	
O1	0.81556 (9)	0.2307 (5)	0.3227 (3)	0.0781 (7)	
C13	0.83539 (15)	0.0345 (9)	0.4189 (5)	0.1010 (15)	
H13A	0.8734	0.0434	0.4432	0.152*	
H13B	0.8257	−0.0970	0.3487	0.152*	
H13C	0.8204	0.0258	0.5292	0.152*	

### (II) 2,5-Bis{[(R)-(-)-1-(4-methoxyphenyl)ethyl]iminomethyl}thiophene Atomic displacement parameters (Å^2^)

**Table:**

	*U*^11^	*U*^22^	*U*^33^	*U*^12^	*U*^13^	*U*^23^
S1	0.0540 (6)	0.0354 (5)	0.0603 (6)	0.000	0.0183 (4)	0.000
N1	0.0442 (13)	0.0512 (16)	0.0477 (13)	0.0014 (10)	0.0129 (10)	0.0058 (10)
C2	0.0460 (15)	0.0443 (18)	0.0507 (16)	−0.0021 (13)	0.0071 (13)	0.0121 (13)
C3	0.0421 (14)	0.0392 (14)	0.0534 (17)	−0.0002 (12)	0.0062 (13)	0.0052 (12)
C4	0.074 (2)	0.0365 (16)	0.071 (2)	0.0007 (13)	0.0193 (16)	0.0074 (13)
C5	0.0497 (16)	0.0537 (18)	0.0434 (15)	0.0015 (12)	0.0120 (12)	0.0097 (12)
C6	0.0626 (19)	0.079 (2)	0.0551 (19)	−0.0010 (18)	0.0058 (15)	−0.0036 (17)
C7	0.0468 (15)	0.0492 (15)	0.0387 (14)	0.0022 (13)	0.0147 (12)	0.0018 (12)
C8	0.0559 (17)	0.0500 (17)	0.0451 (14)	−0.0025 (13)	0.0169 (13)	0.0045 (12)
C9	0.0528 (17)	0.067 (2)	0.0548 (17)	−0.0139 (16)	0.0177 (14)	−0.0004 (16)
C10	0.0455 (16)	0.078 (2)	0.0414 (16)	0.0001 (15)	0.0080 (13)	−0.0080 (15)
C11	0.0575 (18)	0.068 (2)	0.0524 (18)	0.0087 (17)	0.0086 (14)	0.0150 (15)
C12	0.0527 (17)	0.0569 (17)	0.0574 (19)	−0.0031 (14)	0.0138 (14)	0.0147 (15)
O1	0.0478 (13)	0.121 (2)	0.0642 (14)	−0.0021 (14)	0.0011 (10)	−0.0011 (14)
C13	0.063 (2)	0.161 (4)	0.075 (3)	0.022 (3)	−0.0086 (19)	0.022 (3)

### (II) 2,5-Bis{[(R)-(-)-1-(4-methoxyphenyl)ethyl]iminomethyl}thiophene Geometric parameters (Å, º)

**Table:**

S1—C3^i^	1.724 (3)	C7—C12	1.381 (4)
S1—C3	1.724 (3)	C7—C8	1.385 (3)
N1—C2	1.253 (3)	C8—C9	1.374 (4)
N1—C5	1.480 (3)	C8—H8A	0.9300
C2—C3	1.453 (4)	C9—C10	1.379 (4)
C2—H2A	0.9300	C9—H9A	0.9300
C3—C4	1.345 (4)	C10—C11	1.371 (4)
C4—C4^i^	1.413 (6)	C10—O1	1.375 (3)
C4—H4A	0.9300	C11—C12	1.384 (4)
C5—C6	1.504 (4)	C11—H11A	0.9300
C5—C7	1.519 (4)	C12—H12A	0.9300
C5—H5A	0.9800	O1—C13	1.433 (5)
C6—H6A	0.9600	C13—H13A	0.9600
C6—H6B	0.9600	C13—H13B	0.9600
C6—H6C	0.9600	C13—H13C	0.9600
			
C3^i^—S1—C3	91.01 (19)	C12—C7—C5	121.8 (2)
C2—N1—C5	116.9 (2)	C8—C7—C5	120.4 (2)
N1—C2—C3	124.2 (3)	C9—C8—C7	121.2 (3)
N1—C2—H2A	117.9	C9—C8—H8A	119.4
C3—C2—H2A	117.9	C7—C8—H8A	119.4
C4—C3—C2	127.1 (3)	C8—C9—C10	120.1 (3)
C4—C3—S1	111.6 (2)	C8—C9—H9A	119.9
C2—C3—S1	121.3 (2)	C10—C9—H9A	119.9
C3—C4—C4^i^	112.91 (17)	C11—C10—O1	124.7 (3)
C3—C4—H4A	123.5	C11—C10—C9	119.8 (3)
C4^i^—C4—H4A	123.5	O1—C10—C9	115.5 (3)
N1—C5—C6	109.7 (2)	C10—C11—C12	119.7 (3)
N1—C5—C7	108.3 (2)	C10—C11—H11A	120.2
C6—C5—C7	113.7 (2)	C12—C11—H11A	120.2
N1—C5—H5A	108.3	C7—C12—C11	121.5 (3)
C6—C5—H5A	108.3	C7—C12—H12A	119.3
C7—C5—H5A	108.3	C11—C12—H12A	119.3
C5—C6—H6A	109.5	C10—O1—C13	117.0 (3)
C5—C6—H6B	109.5	O1—C13—H13A	109.5
H6A—C6—H6B	109.5	O1—C13—H13B	109.5
C5—C6—H6C	109.5	H13A—C13—H13B	109.5
H6A—C6—H6C	109.5	O1—C13—H13C	109.5
H6B—C6—H6C	109.5	H13A—C13—H13C	109.5
C12—C7—C8	117.8 (3)	H13B—C13—H13C	109.5
			
C5—N1—C2—C3	−175.3 (2)	C12—C7—C8—C9	1.5 (4)
N1—C2—C3—C4	171.0 (3)	C5—C7—C8—C9	−179.4 (3)
N1—C2—C3—S1	−5.7 (4)	C7—C8—C9—C10	−0.3 (4)
C3^i^—S1—C3—C4	−0.22 (17)	C8—C9—C10—C11	−1.1 (4)
C3^i^—S1—C3—C2	176.9 (3)	C8—C9—C10—O1	178.3 (2)
C2—C3—C4—C4^i^	−176.3 (3)	O1—C10—C11—C12	−178.1 (3)
S1—C3—C4—C4^i^	0.6 (4)	C9—C10—C11—C12	1.2 (4)
C2—N1—C5—C6	−136.4 (3)	C8—C7—C12—C11	−1.4 (4)
C2—N1—C5—C7	99.0 (3)	C5—C7—C12—C11	179.6 (3)
N1—C5—C7—C12	63.9 (3)	C10—C11—C12—C7	0.0 (5)
C6—C5—C7—C12	−58.3 (3)	C11—C10—O1—C13	4.8 (4)
N1—C5—C7—C8	−115.0 (3)	C9—C10—O1—C13	−174.6 (3)
C6—C5—C7—C8	122.7 (3)		

Symmetry code: (i) −*x*+1, *y*, −*z*+1.

### (II) 2,5-Bis{[(R)-(-)-1-(4-methoxyphenyl)ethyl]iminomethyl}thiophene Hydrogen-bond geometry (Å, º)

**Table:**

*D*—H···*A*	*D*—H	H···*A*	*D*···*A*	*D*—H···*A*
C4—H4*A*···S1^ii^	0.93	2.99	3.572 (3)	122

Symmetry code: (ii) *x*, *y*+1, *z*.

### (III) 2,5-Bis{[(R)-(-)-1-(4-fluorophenyl)ethyl]iminomethyl}thiophene Crystal data

**Table:**

C_22_H_20_F_2_N_2_S	*D*_x_ = 1.260 Mg m^−^^3^
*M**_r_* = 382.46	Melting point: 420 K
Orthorhombic, *P*2_1_2_1_2	Mo *K*α radiation, λ = 0.71073 Å
*a* = 21.1153 (16) Å	Cell parameters from 2744 reflections
*b* = 7.7846 (6) Å	θ = 3.8–23.2°
*c* = 6.1343 (5) Å	µ = 0.19 mm^−^^1^
*V* = 1008.32 (14) Å^3^	*T* = 298 K
*Z* = 2	Prism, colourless
*F*(000) = 400	0.89 × 0.47 × 0.33 mm

### (III) 2,5-Bis{[(R)-(-)-1-(4-fluorophenyl)ethyl]iminomethyl}thiophene Data collection

**Table:**

Agilent Xcalibur (Atlas, Gemini) diffractometer	2067 independent reflections
Radiation source: Enhance (Mo) X-ray Source	1591 reflections with *I* > 2σ(*I*)
Graphite monochromator	*R*_int_ = 0.058
Detector resolution: 10.5564 pixels mm^-1^	θ_max_ = 26.4°, θ_min_ = 3.8°
ω scans	*h* = −26→26
Absorption correction: analytical *CrysAlis PRO*, (Agilent, 2013)	*k* = −9→9
*T*_min_ = 0.904, *T*_max_ = 0.958	*l* = −7→7
12336 measured reflections	

### (III) 2,5-Bis{[(R)-(-)-1-(4-fluorophenyl)ethyl]iminomethyl}thiophene Refinement

**Table:**

Refinement on *F*^2^	Secondary atom site location: difference Fourier map
Least-squares matrix: full	Hydrogen site location: inferred from neighbouring sites
*R*[*F*^2^ > 2σ(*F*^2^)] = 0.044	H-atom parameters constrained
*wR*(*F*^2^) = 0.092	*w* = 1/[σ^2^(*F*_o_^2^) + (0.0384*P*)^2^ + 0.0613*P*] where *P* = (*F*_o_^2^ + 2*F*_c_^2^)/3
*S* = 1.06	(Δ/σ)_max_ < 0.001
2067 reflections	Δρ_max_ = 0.15 e Å^−^^3^
124 parameters	Δρ_min_ = −0.25 e Å^−^^3^
0 restraints	Absolute structure: Flack *x* determined using 518 quotients [(*I*^+^)-(*I*^-^)]/[(*I*^+^)+(*I*^-^)] (Parsons *et al.*, 2013)
Primary atom site location: structure-invariant direct methods	Absolute structure parameter: 0.07 (6)

### (III) 2,5-Bis{[(R)-(-)-1-(4-fluorophenyl)ethyl]iminomethyl}thiophene Fractional atomic coordinates and isotropic or equivalent isotropic displacement parameters (Å^2^)

**Table:**

	*x*	*y*	*z*	*U*_iso_*/*U*_eq_	
S1	0.5000	0.0000	1.06817 (15)	0.0479 (3)	
F1	0.85802 (10)	0.3203 (3)	0.5731 (5)	0.1163 (9)	
N1	0.58698 (11)	0.3119 (3)	1.0120 (4)	0.0498 (6)	
C2	0.56853 (13)	0.2830 (4)	1.2046 (5)	0.0479 (8)	
H2A	0.5795	0.3610	1.3130	0.057*	
C3	0.53102 (13)	0.1344 (4)	1.2646 (4)	0.0461 (8)	
C4	0.51751 (14)	0.0774 (4)	1.4690 (4)	0.0556 (8)	
H4A	0.5300	0.1342	1.5953	0.067*	
C5	0.62513 (14)	0.4679 (4)	0.9770 (5)	0.0568 (8)	
H5A	0.6336	0.5216	1.1185	0.068*	
C6	0.58661 (16)	0.5931 (5)	0.8368 (7)	0.0829 (12)	
H6A	0.5476	0.6200	0.9094	0.124*	
H6B	0.5777	0.5412	0.6983	0.124*	
H6C	0.6105	0.6967	0.8149	0.124*	
C7	0.68777 (14)	0.4223 (3)	0.8702 (5)	0.0462 (7)	
C8	0.74419 (15)	0.4799 (4)	0.9563 (5)	0.0577 (8)	
H8A	0.7436	0.5427	1.0852	0.069*	
C9	0.80144 (15)	0.4470 (4)	0.8567 (7)	0.0697 (10)	
H9A	0.8391	0.4873	0.9163	0.084*	
C10	0.80134 (17)	0.3551 (5)	0.6707 (7)	0.0689 (10)	
C11	0.74733 (18)	0.2932 (4)	0.5777 (5)	0.0654 (9)	
H11A	0.7489	0.2301	0.4491	0.078*	
C12	0.69042 (15)	0.3265 (4)	0.6789 (5)	0.0543 (8)	
H12A	0.6532	0.2843	0.6184	0.065*	

### (III) 2,5-Bis{[(R)-(-)-1-(4-fluorophenyl)ethyl]iminomethyl}thiophene Atomic displacement parameters (Å^2^)

**Table:**

	*U*^11^	*U*^22^	*U*^33^	*U*^12^	*U*^13^	*U*^23^
S1	0.0499 (6)	0.0559 (6)	0.0379 (5)	−0.0056 (6)	0.000	0.000
F1	0.0687 (14)	0.1167 (19)	0.164 (2)	−0.0047 (14)	0.0485 (15)	−0.030 (2)
N1	0.0452 (14)	0.0502 (15)	0.0540 (16)	−0.0082 (12)	0.0032 (11)	−0.0028 (11)
C2	0.0405 (16)	0.054 (2)	0.0489 (18)	−0.0028 (15)	−0.0043 (14)	−0.0073 (16)
C3	0.0388 (15)	0.0544 (19)	0.0452 (17)	−0.0011 (15)	−0.0013 (13)	−0.0024 (14)
C4	0.056 (2)	0.071 (2)	0.0396 (15)	−0.0115 (15)	−0.0020 (13)	−0.0040 (14)
C5	0.0545 (18)	0.0513 (19)	0.0647 (18)	−0.0104 (16)	0.0112 (15)	−0.0111 (16)
C6	0.065 (2)	0.060 (2)	0.123 (3)	0.0111 (19)	0.023 (2)	0.016 (2)
C7	0.0468 (17)	0.0403 (15)	0.0514 (17)	−0.0063 (14)	0.0002 (14)	0.0001 (13)
C8	0.0576 (19)	0.0492 (17)	0.0662 (19)	−0.0090 (17)	0.0002 (16)	−0.0104 (18)
C9	0.047 (2)	0.064 (2)	0.099 (3)	−0.0114 (17)	−0.0023 (19)	−0.007 (2)
C10	0.054 (2)	0.058 (2)	0.095 (3)	−0.0009 (18)	0.021 (2)	0.001 (2)
C11	0.075 (2)	0.063 (2)	0.0577 (19)	0.000 (2)	0.011 (2)	−0.0053 (18)
C12	0.0499 (18)	0.0573 (19)	0.0557 (18)	−0.0058 (17)	−0.0063 (16)	−0.0020 (16)

### (III) 2,5-Bis{[(R)-(-)-1-(4-fluorophenyl)ethyl]iminomethyl}thiophene Geometric parameters (Å, º)

**Table:**

S1—C3^i^	1.725 (3)	C6—H6A	0.9600
S1—C3	1.725 (3)	C6—H6B	0.9600
F1—C10	1.365 (4)	C6—H6C	0.9600
N1—C2	1.264 (4)	C7—C8	1.378 (4)
N1—C5	1.473 (4)	C7—C12	1.392 (4)
C2—C3	1.450 (4)	C8—C9	1.378 (4)
C2—H2A	0.9300	C8—H8A	0.9300
C3—C4	1.360 (4)	C9—C10	1.347 (5)
C4—C4^i^	1.414 (6)	C9—H9A	0.9300
C4—H4A	0.9300	C10—C11	1.363 (5)
C5—C7	1.518 (4)	C11—C12	1.377 (4)
C5—C6	1.533 (5)	C11—H11A	0.9300
C5—H5A	0.9800	C12—H12A	0.9300
			
C3^i^—S1—C3	91.4 (2)	H6A—C6—H6C	109.5
C2—N1—C5	116.8 (3)	H6B—C6—H6C	109.5
N1—C2—C3	123.2 (3)	C8—C7—C12	117.6 (3)
N1—C2—H2A	118.4	C8—C7—C5	120.8 (3)
C3—C2—H2A	118.4	C12—C7—C5	121.6 (3)
C4—C3—C2	127.5 (3)	C7—C8—C9	121.9 (3)
C4—C3—S1	111.5 (2)	C7—C8—H8A	119.1
C2—C3—S1	120.9 (2)	C9—C8—H8A	119.1
C3—C4—C4^i^	112.81 (18)	C10—C9—C8	118.2 (3)
C3—C4—H4A	123.6	C10—C9—H9A	120.9
C4^i^—C4—H4A	123.6	C8—C9—H9A	120.9
N1—C5—C7	110.3 (2)	C9—C10—C11	122.9 (3)
N1—C5—C6	108.4 (2)	C9—C10—F1	118.4 (3)
C7—C5—C6	111.6 (2)	C11—C10—F1	118.7 (3)
N1—C5—H5A	108.8	C10—C11—C12	118.4 (3)
C7—C5—H5A	108.8	C10—C11—H11A	120.8
C6—C5—H5A	108.8	C12—C11—H11A	120.8
C5—C6—H6A	109.5	C11—C12—C7	121.1 (3)
C5—C6—H6B	109.5	C11—C12—H12A	119.5
H6A—C6—H6B	109.5	C7—C12—H12A	119.5
C5—C6—H6C	109.5		
			
C5—N1—C2—C3	−179.6 (2)	C6—C5—C7—C12	−67.4 (4)
N1—C2—C3—C4	168.6 (3)	C12—C7—C8—C9	1.0 (5)
N1—C2—C3—S1	−8.7 (4)	C5—C7—C8—C9	−176.9 (3)
C3^i^—S1—C3—C4	−0.29 (16)	C7—C8—C9—C10	−0.4 (5)
C3^i^—S1—C3—C2	177.4 (3)	C8—C9—C10—C11	−0.2 (5)
C2—C3—C4—C4^i^	−176.7 (3)	C8—C9—C10—F1	−179.1 (3)
S1—C3—C4—C4^i^	0.8 (4)	C9—C10—C11—C12	0.0 (6)
C2—N1—C5—C7	124.5 (3)	F1—C10—C11—C12	179.0 (3)
C2—N1—C5—C6	−113.0 (3)	C10—C11—C12—C7	0.6 (5)
N1—C5—C7—C8	−129.0 (3)	C8—C7—C12—C11	−1.1 (4)
C6—C5—C7—C8	110.4 (3)	C5—C7—C12—C11	176.7 (3)
N1—C5—C7—C12	53.2 (4)		

Symmetry code: (i) −*x*+1, −*y*, *z*.

### (IV) 2,5-Bis{[(S)-(+)-1-(4-chlorophenyl)ethyl]iminomethyl}thiophene Crystal data

**Table:**

C_22_H_20_Cl_2_N_2_S	*D*_x_ = 1.276 Mg m^−^^3^
*M**_r_* = 415.36	Melting point: 434 K
Orthorhombic, *P*2_1_2_1_2	Mo *K*α radiation, λ = 0.71073 Å
*a* = 21.893 (2) Å	Cell parameters from 2744 reflections
*b* = 7.9212 (6) Å	θ = 3.7–21.5°
*c* = 6.2315 (4) Å	µ = 0.41 mm^−^^1^
*V* = 1080.66 (15) Å^3^	*T* = 298 K
*Z* = 2	Prism, colorless
*F*(000) = 432	0.52 × 0.40 × 0.07 mm

### (IV) 2,5-Bis{[(S)-(+)-1-(4-chlorophenyl)ethyl]iminomethyl}thiophene Data collection

**Table:**

Agilent Xcalibur (Atlas, Gemini) diffractometer	2743 independent reflections
Radiation source: Enhance (Mo) X-ray Source	1534 reflections with *I* > 2σ(*I*)
Graphite monochromator	*R*_int_ = 0.058
Detector resolution: 10.5564 pixels mm^-1^	θ_max_ = 29.5°, θ_min_ = 3.3°
ω scans	*h* = −28→27
Absorption correction: multi-scan *CrysAlis PRO*, (Agilent, 2013)	*k* = −10→9
*T*_min_ = 0.692, *T*_max_ = 1.000	*l* = −8→8
14195 measured reflections	

### (IV) 2,5-Bis{[(S)-(+)-1-(4-chlorophenyl)ethyl]iminomethyl}thiophene Refinement

**Table:**

Refinement on *F*^2^	Secondary atom site location: difference Fourier map
Least-squares matrix: full	Hydrogen site location: inferred from neighbouring sites
*R*[*F*^2^ > 2σ(*F*^2^)] = 0.052	H-atom parameters constrained
*wR*(*F*^2^) = 0.117	*w* = 1/[σ^2^(*F*_o_^2^) + (0.0483*P*)^2^] where *P* = (*F*_o_^2^ + 2*F*_c_^2^)/3
*S* = 1.01	(Δ/σ)_max_ < 0.001
2743 reflections	Δρ_max_ = 0.13 e Å^−^^3^
124 parameters	Δρ_min_ = −0.17 e Å^−^^3^
0 restraints	Absolute structure: Flack *x* determined using 465 quotients [(*I*^+^)-(*I*^-^)]/[(*I*^+^)+(*I*^-^)] (Parsons *et al.*, 2013)
Primary atom site location: structure-invariant direct methods	Absolute structure parameter: 0.10 (6)

### (IV) 2,5-Bis{[(S)-(+)-1-(4-chlorophenyl)ethyl]iminomethyl}thiophene Fractional atomic coordinates and isotropic or equivalent isotropic displacement parameters (Å^2^)

**Table:**

	*x*	*y*	*z*	*U*_iso_*/*U*_eq_	
S1	0.5000	1.0000	−0.13764 (18)	0.0590 (4)	
Cl1	0.15354 (6)	0.67176 (16)	0.4970 (2)	0.1059 (5)	
N1	0.41391 (14)	0.6958 (3)	−0.0839 (5)	0.0612 (8)	
C2	0.43185 (16)	0.7244 (4)	−0.2735 (6)	0.0589 (10)	
H2A	0.4204	0.6496	−0.3812	0.071*	
C3	0.46969 (15)	0.8689 (4)	−0.3311 (5)	0.0544 (9)	
C4	0.48309 (16)	0.9247 (4)	−0.5338 (5)	0.0640 (10)	
H4A	0.4711	0.8688	−0.6581	0.077*	
C5	0.37559 (18)	0.5438 (4)	−0.0520 (7)	0.0677 (11)	
H5A	0.3625	0.5021	−0.1927	0.081*	
C6	0.4148 (2)	0.4087 (5)	0.0553 (9)	0.0986 (17)	
H6A	0.4484	0.3803	−0.0369	0.148*	
H6B	0.3905	0.3098	0.0810	0.148*	
H6C	0.4302	0.4509	0.1892	0.148*	
C7	0.31964 (17)	0.5839 (4)	0.0796 (5)	0.0555 (9)	
C8	0.26313 (19)	0.5202 (5)	0.0255 (7)	0.0701 (10)	
H8A	0.2593	0.4575	−0.0999	0.084*	
C9	0.21198 (19)	0.5464 (5)	0.1512 (7)	0.0735 (11)	
H9A	0.1744	0.5020	0.1109	0.088*	
C10	0.21746 (18)	0.6384 (5)	0.3351 (6)	0.0640 (10)	
C11	0.2728 (2)	0.7062 (5)	0.3945 (6)	0.0691 (10)	
H11A	0.2761	0.7692	0.5199	0.083*	
C12	0.32346 (18)	0.6802 (4)	0.2665 (5)	0.0623 (9)	
H12A	0.3607	0.7276	0.3056	0.075*	

### (IV) 2,5-Bis{[(S)-(+)-1-(4-chlorophenyl)ethyl]iminomethyl}thiophene Atomic displacement parameters (Å^2^)

**Table:**

	*U*^11^	*U*^22^	*U*^33^	*U*^12^	*U*^13^	*U*^23^
S1	0.0694 (9)	0.0596 (7)	0.0482 (6)	−0.0040 (7)	0.000	0.000
Cl1	0.0878 (9)	0.1061 (9)	0.1238 (10)	0.0069 (7)	0.0384 (8)	0.0026 (9)
N1	0.062 (2)	0.0569 (18)	0.0643 (19)	−0.0089 (15)	0.0064 (15)	0.0009 (14)
C2	0.055 (2)	0.060 (2)	0.061 (2)	0.0015 (17)	−0.0031 (19)	−0.0056 (18)
C3	0.050 (2)	0.060 (2)	0.0523 (19)	0.0004 (17)	−0.0008 (16)	−0.0008 (17)
C4	0.063 (3)	0.080 (2)	0.0487 (18)	−0.0130 (18)	−0.0011 (17)	−0.0059 (17)
C5	0.071 (3)	0.057 (2)	0.075 (2)	−0.0098 (18)	0.015 (2)	−0.0050 (18)
C6	0.085 (3)	0.064 (2)	0.147 (4)	0.011 (2)	0.040 (3)	0.019 (3)
C7	0.061 (2)	0.0442 (17)	0.061 (2)	−0.0052 (17)	−0.0039 (18)	0.0020 (16)
C8	0.073 (3)	0.061 (2)	0.076 (2)	−0.017 (2)	−0.002 (2)	−0.014 (2)
C9	0.063 (3)	0.067 (3)	0.091 (3)	−0.0168 (19)	−0.003 (2)	−0.007 (2)
C10	0.065 (3)	0.056 (2)	0.072 (2)	0.0010 (19)	0.007 (2)	0.007 (2)
C11	0.081 (3)	0.068 (2)	0.059 (2)	−0.002 (2)	−0.003 (2)	−0.0067 (18)
C12	0.059 (2)	0.065 (2)	0.063 (2)	−0.004 (2)	−0.010 (2)	−0.002 (2)

### (IV) 2,5-Bis{[(S)-(+)-1-(4-chlorophenyl)ethyl]iminomethyl}thiophene Geometric parameters (Å, º)

**Table:**

S1—C3	1.724 (3)	C6—H6A	0.9600
S1—C3^i^	1.724 (3)	C6—H6B	0.9600
Cl1—C10	1.746 (4)	C6—H6C	0.9600
N1—C2	1.265 (4)	C7—C8	1.378 (5)
N1—C5	1.481 (4)	C7—C12	1.394 (4)
C2—C3	1.458 (5)	C8—C9	1.382 (5)
C2—H2A	0.9300	C8—H8A	0.9300
C3—C4	1.370 (4)	C9—C10	1.363 (5)
C4—C4^i^	1.404 (7)	C9—H9A	0.9300
C4—H4A	0.9300	C10—C11	1.376 (5)
C5—C7	1.508 (5)	C11—C12	1.382 (5)
C5—C6	1.527 (5)	C11—H11A	0.9300
C5—H5A	0.9800	C12—H12A	0.9300
			
C3—S1—C3^i^	91.3 (2)	H6A—C6—H6C	109.5
C2—N1—C5	116.6 (3)	H6B—C6—H6C	109.5
N1—C2—C3	123.2 (3)	C8—C7—C12	117.3 (4)
N1—C2—H2A	118.4	C8—C7—C5	121.3 (3)
C3—C2—H2A	118.4	C12—C7—C5	121.4 (3)
C4—C3—C2	127.0 (3)	C7—C8—C9	122.2 (4)
C4—C3—S1	111.6 (3)	C7—C8—H8A	118.9
C2—C3—S1	121.3 (2)	C9—C8—H8A	118.9
C3—C4—C4^i^	112.8 (2)	C10—C9—C8	119.0 (4)
C3—C4—H4A	123.6	C10—C9—H9A	120.5
C4^i^—C4—H4A	123.6	C8—C9—H9A	120.5
N1—C5—C7	111.2 (3)	C9—C10—C11	120.8 (4)
N1—C5—C6	108.1 (3)	C9—C10—Cl1	119.8 (3)
C7—C5—C6	111.5 (3)	C11—C10—Cl1	119.4 (3)
N1—C5—H5A	108.7	C10—C11—C12	119.5 (3)
C7—C5—H5A	108.7	C10—C11—H11A	120.2
C6—C5—H5A	108.7	C12—C11—H11A	120.2
C5—C6—H6A	109.5	C11—C12—C7	121.0 (4)
C5—C6—H6B	109.5	C11—C12—H12A	119.5
H6A—C6—H6B	109.5	C7—C12—H12A	119.5
C5—C6—H6C	109.5		
			
C5—N1—C2—C3	179.8 (3)	C6—C5—C7—C12	74.9 (4)
N1—C2—C3—C4	−168.8 (4)	C12—C7—C8—C9	−1.2 (5)
N1—C2—C3—S1	7.3 (5)	C5—C7—C8—C9	175.8 (3)
C3^i^—S1—C3—C4	0.42 (19)	C7—C8—C9—C10	0.0 (6)
C3^i^—S1—C3—C2	−176.3 (4)	C8—C9—C10—C11	0.8 (6)
C2—C3—C4—C4^i^	175.3 (4)	C8—C9—C10—Cl1	−179.6 (3)
S1—C3—C4—C4^i^	−1.1 (5)	C9—C10—C11—C12	−0.3 (6)
C2—N1—C5—C7	−132.2 (3)	Cl1—C10—C11—C12	−179.9 (3)
C2—N1—C5—C6	105.1 (4)	C10—C11—C12—C7	−1.0 (6)
N1—C5—C7—C8	137.3 (4)	C8—C7—C12—C11	1.7 (5)
C6—C5—C7—C8	−102.0 (4)	C5—C7—C12—C11	−175.3 (3)
N1—C5—C7—C12	−45.8 (4)		

Symmetry code: (i) −*x*+1, −*y*+2, *z*.
